# Learning by motor imagery in older adults

**DOI:** 10.18632/aging.205185

**Published:** 2023-10-13

**Authors:** Arnaud Saimpont, Angèle Métais, Christian Collet

**Affiliations:** 1 Université Lyon, UCBL-Lyon 1, Laboratoire Interuniversitaire de Biologie de la Motricité, UR 7424, France

**Keywords:** motor imagery, motor sequence learning (MSL), tDCS, aging

Functional motor skills are crucial to keep a good quality of life in older adults. While motor learning/relearning may be relatively preserved with aging, it typically requires repetitive practice, which can be challenging. This challenge becomes even more important when considering rehabilitation protocols for older individuals recovering from stroke, falls, or hip surgery, as they often experience physical fatigue. Therefore, there is a need to promote reliable interventions, simple to implement, and less physically demanding, with the aim of preserving or even improving motor functions in the elderly population.

Among different options, there has been a growing interest in motor imagery (MI) over the past two decades. MI refers to the mental simulation of a motor action without actual physical output. It is often performed from a first-person perspective, where individuals mentally perceive (visually and/or kinesthetically) what they would normally perceive during the actual execution of the movement. Numerous investigations have provided evidence that MI positively influences motor learning and recovery across various populations. While inter-individual variability is high, overall MI ability remains preserved until around 80 years, given intact working memory [1]. In fact, some studies have shown that MI contributes to improve motor performance in older adults [2]. A noteworthy finding was that twelve sessions of six minutes of MI over six weeks yielded beneficial effects on a sequential finger tapping task (SFTT) [3]. In this study, the number of finger movements significantly increased and remained stable, even one week after training. However, specific information regarding online and offline gains during learning was not provided. Indeed, if motor sequence learning (MSL) ultimately refers to the retention of movement sequences in long-term memory, it is possible to distinguish two sub-processes during MSL. First, online learning, where performance improves through practice – especially during the first training session. Second, offline learning, where performance may be consolidated (stabilized or even improved) during the hours following training, without additional practice. MSL is selectively affected in adults over 65 years of age, online gains are relatively preserved but performance may decline during the consolidation process [4]. Whether these sub-processes are similarly or differently affected by aging when learning occurs through MI remained unexplored.

Regardless of the studies on MI in the elderly, it has been shown that anodal transcranial direct current stimulation (a-tDCS), involving the application of a weak current (1–2 mA) between an anode and a cathode placed on the scalp, may enhance MSL in older adults. For example, the combination of physical practice with a-tDCS applied to the primary motor cortex over five consecutive days of training resulted in greater online effects (compared to sham stimulation) on implicit MSL, in the second, third and fourth days [5]. Also, three consecutive sessions of a-tDCS over the primary motor cortex, in conjunction with either physical or MI practice, yielded improvements in implicit MSL in younger adults [6]. Specifically, the online gains through MI practice were greater during the first training session with a-tDCS. Additionally, there was a consolidation of performance in the MI groups, although it was not enhanced by a-tDCS, indicating the absence of offline effects of stimulation. Lastly, it is worth mentioning that the benefits of a-tDCS were more pronounced after three training sessions compared to a single session, irrespective of the practice groups. Whether a-tDCS could also improve the online gains of MI practice in older adults remained untested.

We thus conducted a randomized controlled study aimed to investigate the cumulative effects of three consecutive daily sessions of MI in combination with either a-tDCS (active group) or sham tDCS (sham group) over the primary motor cortex, on learning a SFTT, in healthy older adults [7]. Both groups exhibited cumulative online gains over the course of the first two training sessions, with greater improvements in the first session compared to the second, and no additional gains during the third session. This pattern of performance evolution over time is similar to what observed in several physical practice sessions of a SFTT among older adults [8]. Additionally, performance remained stable overnight between all training sessions, indicating that the memory trace established through MI training was consolidated offline, without further practice. Furthermore, performance remained unchanged one week after the final training session. Although the retention period studied in our research was relatively shorter, this finding is also consistent with the stabilization of performance observed one month after several sessions of practice reported by Gal et al. (2019) [8]. However, contrary to our expectations, the combination of multiple sessions of a-tDCS with MI did not enhance performance gains. In fact, findings on tDCS effects on motor learning are inconclusive and vary among studies. Regarding our experiment, among other factors, it is possible that the intensity of stimulation (1.5 mA) was too low to promote cortical plasticity during learning by MI.

To sum up, we showed that three consecutive daily sessions of MI led to performance improvements in a SFTT, primarily driven by online effects observed during the first two training sessions. Additionally, we observed a stabilization of performance between training sessions (offline consolidation) and long-term retention one week after training. This work emphasizes the importance of repeatedly practicing MI to maximize its effect in older adults. However, as already demonstrated in young adults, we recently confirmed that combining MI to physical practice led to similar outcomes than physical practice alone in the elderly (unpublished data). If an older individual is capable of making a movement but finds it taxing, the combination of physical and MI practice appears the best way to use MI. Finally, how combining anodal tDCS with MI in the elderly, including questions about where, how, and when stimulation should be applied, is one of the next challenges in this field of research.
